# Vedolizumab: Potential Mechanisms of Action for Reducing Pathological Inflammation in Inflammatory Bowel Diseases

**DOI:** 10.3389/fcell.2021.612830

**Published:** 2021-02-03

**Authors:** Matthew Luzentales-Simpson, Yvonne C. F. Pang, Ada Zhang, James A. Sousa, Laura M. Sly

**Affiliations:** Division of Gastroenterology, Department of Pediatrics, BC Children’s Hospital and the University of British Columbia, Vancouver, BC, Canada

**Keywords:** vedolizumab, inflammatory bowel disease, cell trafficking, macrophages, innate immunity

## Abstract

Inflammatory bowel diseases (IBD), encompassing ulcerative colitis (UC), and Crohn’s disease (CD), are a group of disorders characterized by chronic, relapsing, and remitting, or progressive inflammation along the gastrointestinal tract. IBD is accompanied by massive infiltration of circulating leukocytes into the intestinal mucosa. Leukocytes such as neutrophils, monocytes, and T-cells are recruited to the affected site, exacerbating inflammation and causing tissue damage. Current treatments used to block inflammation in IBD include aminosalicylates, corticosteroids, immunosuppressants, and biologics. The first successful biologic, which revolutionized IBD treatment, targeted the pro-inflammatory cytokine, tumor necrosis factor alpha (TNFα). Infliximab, adalimumab, and other anti-TNF antibodies neutralize TNFα, preventing interactions with its receptors and reducing the inflammatory response. However, up to 40% of people with IBD become unresponsive to anti-TNFα therapy. Thus, more recent biologics have been designed to block leukocyte trafficking to the inflamed intestine by targeting integrins and adhesins. For example, natalizumab targets the α4 chain of integrin heterodimers, α4β1 and α4β7, on leukocytes. However, binding of α4β1 is associated with increased risk for developing progressive multifocal leukoencephalopathy, an often-fatal disease, and thus, it is not used to treat IBD. To target leukocyte infiltration without this life-threatening complication, vedolizumab was developed. Vedolizumab specifically targets the α4β7 integrin and was approved to treat IBD based on the presumption that it would block T-cell recruitment to the intestine. Though vedolizumab is an effective treatment for IBD, some studies suggest that it may not block T-cell recruitment to the intestine and its mechanism(s) of action remain unclear. Vedolizumab may reduce inflammation by blocking recruitment of T-cells, or pro-inflammatory monocytes and dendritic cells to the intestine, and/or vedolizumab may lead to changes in the programming of innate and acquired immune cells dampening down inflammation.

## Introduction

Inflammatory bowel disease (IBD), encompassing ulcerative colitis (UC), and Crohn’s disease (CD) are a group of disorders that are characterized by chronic, relapsing, and remitting, or progressive inflammation along the gastrointestinal tract. UC involves continuous inflammation of the colon and the rectum (Fakhoury et al., 2014). It is limited to the mucosal layer of the intestinal wall. In contrast, CD causes discontinuous, transmural inflammation that can occur anywhere along the digestive tract, though it most commonly affects the distal ileum (Fakhoury et al., 2014). Canada has among the highest prevalence of IBD with 1 in 140 people affected (Kaplan et al., 2019). IBD has traditionally been regarded as a disease of developed and high-income nations, though incidence rates appear to have stabilized with high burden and prevalence. Incidence of IBD is now rapidly rising in newly industrialized countries (GBD 2017 Inflammatory Bowel Disease Collaborators, 2020). There were 6.8 million cases of IBD globally in 2017, with approximately 1.5 million in North America and 2 million in Europe (GBD 2017 Inflammatory Bowel Disease Collaborators, 2020; Jairath and Feagan, 2020). Individuals with IBD suffer from intestinal inflammation that can lead to debilitating symptoms including pain, nausea, and diarrhea (Rosen et al., 2015). In addition to disease burden, the chronic nature of IBD and its requirement for lifelong treatment causes significant economic burden for the individual and society. As analyzed by Park et al. (2020), the annual mean health care cost for people with IBD is over 3-fold higher than for people without IBD (selected from the general population using a health plan member database). Mehta (2016) estimated that extrapolated direct costs of IBD are between $11 to 28 billion in the United States. Including direct and indirect costs associated with loss of productivity and earnings, the total cost of IBD is between $14.6 and 31.6 billion annually (Mehta, 2016).

The etiology of IBD is multifactorial and includes genetic susceptibility, inappropriate immune activity, and environmental triggers. For example, a loss of function gene variant in the NOD2 gene is associated with increased susceptibility to CD due to increased production of pro-inflammatory cytokines (Ogura et al., 2001). Environmental factors affecting incidence of disease include geography, smoking, and pollution (Ananthakrishnan et al., 2018). Genetics, immune responses, and environmental variables also impact the host intestinal microbiota, which is often included as a fourth important factor in the development of IBD (Ananthakrishnan et al., 2018). While the exact cause is unknown, it is widely accepted that IBD occurs in genetically susceptible individuals with environmental influences that result in a dysregulated immune response to commensal intestinal microbiota (Rosen et al., 2015). The wide variation in disease presentation and treatment efficacy reflects the complexity of IBD pathogenesis (Chichlowski and Hale, 2008).

In this review, we will focus on the role of specific immune cells in IBD pathogenesis and how their trafficking and activity may be affected be vedolizumab. IBD is characterized by massive infiltration of circulating leukocytes into the inflamed intestinal mucosa. Diseased sections of the intestines have cytokine profiles that differ from healthy sections, indicating that cytokines play a pivotal role in the incidence and progression of disease (Murch et al., 1993; McAlindon et al., 1998). In particular, immune cells that are isolated from people with IBD display increased expression of pro-inflammatory cytokines and chemokines (Singh et al., 2016). Chemokine production leads to inappropriate recruitment and retention of cells such as T-cells, dendritic cells, and macrophages, which cause inflammation and establish the chronic inflammation that is characteristic of IBD (Singh et al., 2016). Chronic activation and proliferation of these immune cells leads to disruption of healthy tissues and thus further exacerbation of disease (Schippers et al., 2016). Macrophages in particular have a unique role in IBD due to their ability to exhibit pro-inflammatory activity that contributes to disease and Interleukin-10 (IL-10)-mediated anti-inflammatory activity (Kozicky et al., 2015). Anti-inflammatory macrophage activity has recently been described in the resolution of IBD during anti-TNFα therapies in mice and humans (Koelink et al., 2020). Specifically, macrophage IL-10 signaling was described to be the driving force behind the therapeutic effect of these therapies (Koelink et al., 2020). With respect to these studies, IBD therapies can reduce inflammation by targeting leukocyte trafficking to the intestine and pro-inflammatory activity.

There is no standard treatment for IBD. Therapeutic options are non-specific anti-inflammatories that include aminosalicylates, corticosteroids, and other immunosuppressants, and some biologics. New biological therapies have been designed to be specific to the gut to minimize side effects and increase responsiveness (Hazel and O’Connor, 2020). In particular, anti-integrin antibodies target key players in leukocyte trafficking to the gut to reduce immune cell infiltration and the resulting inflammation (Hazel and O’Connor, 2020). Vedolizumab is a therapy that was designed to reduce pathological inflammation in IBD by blocking T-cell recruitment to the intestine (Zeissig et al., 2019). However, some evidence suggests that it does not affect T-cell migration to the intestine (Zeissig et al., 2019). Herein, we describe the potential mechanisms of action by which vedolizumab may reduce pathological inflammation in IBD.

## Leukocyte Trafficking and Activation in IBD Pathogenesis

### Leukocyte Trafficking

Inflammatory bowel diseases is characterized by immune infiltration from the circulation into the inflamed intestinal mucosa. This migration is facilitated by complex interactions between circulating leukocytes and intestinal endothelial cells. Sialyl LewisX-modified glycoproteins on leukocytes bind selectins on the endothelial cells with low affinity, allowing leukocytes to roll along the endothelium (Wright and Cooper, 2014). Chemokines act as chemoattractants for the rolling leukocytes to promote their infiltration into the mucosa (Wright and Cooper, 2014). Integrins on leukocytes mediate adhesion by binding cellular adhesion molecules (CAMs) that are expressed on the endothelial cells during inflammation (Wright and Cooper, 2014). Integrins are heterodimeric receptors composed of an α and a β subunit, which exist in several forms that can combine to allow tissue-specific adhesion (Arseneau and Cominelli, 2015). For example, the α4β7 receptor is a marker for leukocyte trafficking to the intestine (Clahsen et al., 2015).

Three CAMs have been reported to mediate leukocyte trafficking to the inflamed intestine, contributing to IBD: intracellular adhesion molecule (ICAM)-1, mucosal addressin cellular adhesion molecule (MAdCAM)-1, and vascular cell adhesion molecule (VCAM)-1 (Arseneau and Cominelli, 2015). Pro-inflammatory cytokines that are expressed during inflammation increase ICAM-1 expression, which binds the αLβ2 receptor on leukocytes (Bendjelloul et al., 2000; Arseneau and Cominelli, 2015). MAdCAM-1, which is expressed within Peyer’s patches and intestinal lymphoid tissues, binds the α4β7 receptor on memory/effector T-cells to regulate their homing to the intestine (DeNucci et al., 2010; Arseneau and Cominelli, 2015). It is upregulated at sites of inflammation in people with IBD (Briskin et al., 1997). VCAM-1 binds the α4β1 and α4β7 receptors, which mediate leukocyte trafficking to the central nervous system and intestine, respectively, (Arseneau and Cominelli, 2015; Schippers et al., 2016). Blocking the integrin-CAM interactions in the intestine is used as a strategy for IBD therapy aimed at reducing immune cell infiltration into the inflamed intestine.

### T-Cells

T-cells play a prominent role in the regulation of the inflammatory response associated with IBD. In the past, CD was thought to be a Th1-mediated disease characterized by elevated levels of the pro-inflammatory mediators interleukin-2 (IL-2), interferon gamma (IFNγ), and tumor necrosis factor alpha (TNFα; Fais et al., 1991; Breese et al., 1993; Fuss et al., 1996). Since then, additional cytokines have been implicated in the pathogenesis of CD. Neurath et al. (1995) reported that antibodies against the p40 subunit of IL-12, a cytokine which induces Th1 cell differentiation, were able to attenuate 2,4,6-trinitrobenzene sulfonic acid (TNBS)-induced colitis (Jacobson et al., 1995; Neurath et al., 1995). Th17 cells were implicated in intestinal inflammation when IL-23, which maintains and expands Th17 populations, was found to share the p40 subunit with IL-12 (Oppmann et al., 2000; Stritesky et al., 2008). Furthermore, IL-23 was also shown to be a key driver of intestinal inflammation in the *Helicobacter hepaticus* infection and T-cell transfer models of colitis (Hue et al., 2006). UC is characterized by higher expression of IL-5 and IL-13, but not IL-4 (Karttunnen et al., 1994; Fuss et al., 1996). IL-13 is a key effector, synergizing with TNFα to modulate the proteins in tight junction formation, thereby disrupting the epithelial barrier (Heller et al., 2005; Chen and Sundrud, 2016). Recently, Rosen et al. (2017) showed that *IL-17A* and *IL-23* mRNA in pediatric rectal mucosal samples were increased in UC in addition to higher *IL-5* and *IL-13* mRNA. Thus, targeting T-cell trafficking may reduce the relative concentration of proinflammatory cytokines described to be involved in IBD. Finally, T-regulatory (Treg) cells regulate self-reactive lymphocytes by secreting inhibitory cytokines such as IL-10 and transforming growth factor-β (TGFβ; Taylor et al., 2006). By suppressing immune responses and maintaining tolerance to commensal microbes, Tregs are involved in intestinal homeostasis (Himmel et al., 2012).

Recent developments show multiple IBD susceptibility loci associated with T-cell activation and memory formation (Liu et al., 2015). Genes such as CD28 (T-cell co-stimulation), CCL20 and CCR6 (T-cell migration), NFATC1 (lymphocyte proliferation), NFKBIZ (Th17 development) associated with T-cell function demonstrate the potential for therapeutic strategies, which target different stages of T-cell involvement in IBD, such as recruitment, activation, proliferation, and retention. Genome-Wide Association Studies (GWAS) are crucial to explore potential genes associated with disease susceptibility and are further supported by literature that shows protein-level discrepancies between people with IBD and healthy control study participants. The presence of T-cells in the gut of people with IBD may be mediated *via* CCR6, CCL20, or the α4β7 integrin (Perez-Jeldres et al., 2019). Thus, blocking the interaction of these molecules with their respective ligands or receptors has not been accepted as the sole mechanism of T-cell trafficking. Drugs such as natalizumab, which binds α4 integrin, and vedolizumab, which targets the α4β7 heterodimer, have been developed specifically to target T-cell trafficking (Hazel and O’Connor, 2020). Therefore, it is crucial that we continue to explore the potential mechanisms of these, and other, therapies in order to improve existing therapies and to develop new ones.

### Dendritic Cells

Dendritic cells (DCs) are professional antigen-presenting cells that control the innate and adaptive immune responses. In the intestine, there are two described subsets: conventional (cDCs) and plasmacytoid DCs (pDCs). Depending on their location within the epithelium, cDCs either secrete IL-10 and induce Th2 cells or secrete IL-12 and induce Th1 cells (Guan, 2019). They can be further distinguished by their expression of cell surface receptors, such as the integrin subunit CD103 (αE), which binds β7 to form the αEβ7 complex (Johansson-Lindbom et al., 2005; Clahsen et al., 2015). CD103 facilitates the retention of lymphocytes in the epithelium by binding E-cadherin (Johansson-Lindbom et al., 2005). CD103+ cDCs make up the majority of the DC population in the small intestine (Johansson-Lindbom et al., 2005; Clahsen et al., 2015). They are located in the lamina propria and intraepithelial compartment, but they can migrate to the mesenteric lymph node (MLN) to induce expression of the gut homing receptors CCR9 and α4β7 integrin on B and T-cells (Annacker et al., 2005; Schulz et al., 2009). Additionally, CD103+ cDCs can promote the development of Treg cells (Annacker et al., 2005; Clahsen et al., 2015). In contrast, CD103- (CX3CRI+) cDCs do not migrate (Schulz et al., 2009). Instead, they penetrate the epithelium to sample antigens in the lumen and present antigen to CD4+ T-cells, which differentiate into effector T-cells that secrete pro-inflammatory cytokines (Guan, 2019). Finally, pDCs are rare cells that secrete large quantities of type I interferons (Guan, 2019).

During IBD, DCs are attracted to sites of inflammation in the intestine by chemokines, such as CCL20 and MAdCAM-1 (Guan, 2019). Large numbers of activated DCs are found in the lamina propria and MLN and promote inflammation in murine models of IBD (Berndt et al., 2007; Guan, 2019). Conversely, ablation of DCs can also exacerbate disease (Muzaki et al., 2016). By regulating immune responses and tolerance to the microbiota, DCs play a critical role in IBD pathogenesis.

### Macrophages

Macrophages play a role in all stages of inflammation: recognition, response, and resolution. They are highly heterogeneous, demonstrating a wide range of activation states (Kozicky et al., 2015). Cues from the microenvironment polarize macrophages to specific activation states (Kozicky et al., 2015). The three main phenotypes are grouped by function: inflammatory, wound-healing, and regulatory/anti-inflammatory, as reviewed in Steinbach and Plevy (2014). Inflammatory macrophages are promoted by IFNγ from NK and T-helper 1 (Th1) cells and TNFα from antigen presenting cells (Steinbach and Plevy, 2014). TNFα can also result from innate immune stimuli signaling that activates suppressor of cytokine signaling 3 (Steinbach and Plevy, 2014). Inflammatory macrophages produce high levels of the pro-inflammatory cytokines TNFα, IL-12, IL-6, and reactive oxygen and nitrogen species, which promote Th1 and Th17 cell activity and low levels of the anti-inflammatory cytokine, IL-10 (Hausmann et al., 2001; Heinsbroek and Gordon, 2009). Inflammatory macrophages are essential in the response to intracellular infections but can aggravate IBD due to their production of pro-inflammatory cytokines (Heinsbroek and Gordon, 2009). Wound-healing macrophages are induced by IL-4 from granulocytes or Th2 cells in response to tissue injury (Kozicky et al., 2015). They produce relatively lower levels of pro-inflammatory cytokines and higher levels of IL-10, protecting against parasites and promoting wound healing through the suppression of NLRP3 inflammasome activation, angiogenesis, tissue remodeling, and debris scavenging (Steinbach and Plevy, 2014; Yao et al., 2015). Wound-healing macrophages are protective in murine models of intestinal inflammation but may contribute to fibrosis in CD (Steinbach and Plevy, 2014). Regulatory or anti-inflammatory macrophages are a recently described phenotype that require two stimuli, one of which is pro-inflammatory (Mosser and Edwards, 2008; Kozicky et al., 2015). They can be activated by macrophage-derived TGFβ, IL-10, or immune complexes and a pro-inflammatory stimulus (Anderson et al., 2002; Mosser and Edwards, 2008; Kozicky et al., 2015). They produce high levels of IL-10 (Kozicky et al., 2015). In addition, regulatory macrophages express costimulatory molecules that activate T-cells (Steinbach and Plevy, 2014). They further differ from wound-healing macrophages in their lack of extracellular matrix production (Steinbach and Plevy, 2014). Regulatory macrophages play a key role in turning off the inflammatory response by reducing IL-12 synthesis (Kozicky et al., 2019) and are not predicted to promote fibrosis (Steinbach and Plevy, 2014).

In the lamina propria, macrophages control homeostasis by responding to infectious challenges with phagocytic and microbicidal activity while maintaining immune tolerance to commensal microbes. Differentiation into a tolerant phenotype is promoted by the presence of IL-10 and TFGβ in the microenvironment (Smythies et al., 2005). In contrast to the resident macrophages that do not mount an oxidative burst or inflammatory response, circulating blood monocytes are recruited to the sites of inflammation in the intestinal epithelium *via* chemokines and aggravate disease (Guan, 2019). Infiltration of these blood monocytes to local tissues is facilitated through tight α4β7-MAdCAM-1 interactions among other adhesion molecule and cadherin interactions (Berlin et al., 1993; Gorfu et al., 2009). Acute intestinal inflammation and chronic inflammation cause an influx of pro-inflammatory blood monocytes, which differentiate into inflammatory macrophages and exacerbate disease (Schippers et al., 2016). Macrophages isolated from people with IBD have increased oxidative burst activity and pro-inflammatory cytokine production (Guan, 2019). However, invading monocytes have been shown to dampen the inflammatory response through the release of IL-10 (Koelink et al., 2020). There is growing evidence that intestinal macrophages may play a critical role in the resolution of IBD, especially when activated towards regulatory or wound-healing phenotypes (Koelink et al., 2020). For this reason, macrophages, and subcellular molecules that modulate macrophages like chemokines and cytokines are potential therapeutic targets to ameliorate intestinal inflammation in IBD.

## Current Treatments

The increasing disease burden of IBD reflects a need for a greater understanding of the mechanisms of IBD pathogenesis to improve existing therapies and develop new and effective therapies. IBD typically causes significant morbidity and requires lifelong medication in addition to possible dietary and lifestyle changes (Seyedian et al., 2019). There is no standard treatment regimen for individuals with IBD. Therapy relies on non-specific immune suppression to reduce symptoms, maintain remission, and prevent relapse (Hazel and O’Connor, 2020). Therapies include aminosalicylates, non-specific immunosuppressants, steroids, and biologics that target pro-inflammatory cytokines or leukocyte trafficking to the gut (Shi and Ng, 2018; Seyedian et al., 2019; Hazel and O’Connor, 2020). Surgery, which involves removal of the affected region, is an option for some people with acute, severe, refractory UC (Sica and Biancone, 2013), but not CD (Seyedian et al., 2019). Efficacy of therapy can be limited by a lack of primary response, secondary loss of response, and adverse side effects (Shi and Ng, 2018).

### Anti-TNFα Biologics

Tumor necrosis factor alpha is an inflammatory cytokine that plays a prominent role in active inflammation associated with IBD (Fakhoury et al., 2014). An increase in TNFα has been shown to induce cell proliferation, differentiation, and upregulation of adhesion molecules on the endothelium to increase cell trafficking to the site of inflammation (Nawroth and Stern, 1986; Fakhoury et al., 2014). Considering its role in IBD, neutralizing TNFα has been used effectively to treat IBD (mechanisms of action reviewed by Park and Jeen (2015) and Adegbola et al. (2018)).

Anti-TNFα biologics, including golimumab, certolizumab, infliximab, and adalimumab, have revolutionized IBD treatment. However, many people with IBD are unresponsive or experience significant adverse effects; up to 40% of people with IBD are predicted to become unresponsive to anti-TNFα antibodies (Shi and Ng, 2018). More specifically, a recent meta-analysis showed secondary loss of response occurs in approximately 33% of people taking infliximab and 41% of people taking adalimumab with a median follow up of 1 year (Qiu et al., 2017). These studies highlight the need for developing new therapeutics to treat IBD.

### Anti-interleukin Biologics

Ustekinumab is a monoclonal antibody which binds the p40 subunit of IL-12 and IL-23 and acts as an antagonist (Feagan et al., 2016; Sands et al., 2019). People with CD who received ustekinumab had significantly higher clinical response rates in the UNITI-1 and UNITI-2 clinical trials compared to placebo (Feagan et al., 2016). People with UC who were treated with ustekinumab had significantly higher rates of achieving and maintaining clinical remission compared to placebo (Sands et al., 2019). The FDA approved ustekinumab for treatment of moderate to severe CD in September 2016, and for treatment of moderate to severe UC in October 2019.

### Anti-integrin Biologics

#### Natalizumab

Natalizumab is a humanized monoclonal antibody that targets the α4 chain of the α4β1 and α4β7 integrins expressed on the surface of leukocytes (Leger et al., 1997; Stuve et al., 2006; Haanstra et al., 2013). α4β1 binds VCAM-1 expressed by endothelial cells, which allows leukocytes to firmly adhere to the surface (Alon et al., 1995; Cerutti and Ridley, 2017; Chae et al., 2018). Natalizumab blocks the interaction between α4β1 and VCAM-1, which is required for leukocytes to cross the blood-brain barrier into the central nervous system (CNS; Kumar et al., 1975; Burkly et al., 1991).

Natalizumab was first used successfully for the treatment of relapsing and remitting multiple sclerosis (MS; Polman et al., 2006; Rudick et al., 2006). In January 2008, natalizumab was also approved by the United States Food and Drug Administration (FDA) for treatment of CD (Guagnozzi and Caprilli, 2008). It was the first anti-adhesion biologic used to treat IBD and established evidence for the potential efficacy of blocking leukocyte trafficking to treat IBD. However, clinical trials and market distribution of natalizumab were discontinued due to reports that two people had developed progressive multifocal leukoencephalopathy (PML; Kleinschmidt-DeMasters and Tyler, 2005; Langer-Gould et al., 2005; Van Assche et al., 2005). PML is an aggressive demyelinating disease of the central nervous system (CNS) caused by the opportunistic John Cunningham (JC) virus, for which the majority of the population (75–80%) is seropositive. The recall of natalizumab prompted the retrospective analysis of samples from a deceased person with CD who had been treated with natalizumab in a separate clinical trial (Van Assche et al., 2005). The individual’s serum and brain lesion samples were positive for JC virus, and there was a temporal relationship between natalizumab treatment and increase in viral load (Van Assche et al., 2005). Natalizumab was reapproved by the FDA and European Medicine Agencies (EMA) for the treatment of relapsing-remitting MS but it is not used for CD, due to risk of serious infections (European Medicines Agency, 2007; Ransohoff, 2007; Planas et al., 2014; Avasarala, 2015).

#### Vedolizumab

Vedolizumab is a humanized monoclonal antibody that was developed to reduce lymphocyte trafficking to the intestine by specifically targeting the α4β7 heterodimer, which is expressed on the surface of gut-specific lymphocytes (Soler et al., 2009; Feagan et al., 2013). In contrast to natalizumab, vedolizumab does not interfere with lymphocyte trafficking to the brain (Feagan et al., 2013).

Three Phase 3 clinical trials evaluated the efficacy and safety of vedolizumab for the induction and maintenance of clinical response and remission in people with moderate to severe UC (GEMINI 1) and CD (GEMINI 2 and GEMINI 3; Feagan et al., 2013; Sandborn et al., 2013; Sands et al., 2014). In the GEMINI 1 trial, 47.1% of people with UC had clinical responses by week 6 compared to 25.5% on placebo (Feagan et al., 2013). For the maintenance arm, 41.8% of people with UC who were treated with vedolizumab every 8 weeks and 44.8% of people with UC who were treated every 4 weeks maintained clinical remission at week 52 of the trial compared to only 15.9% of people with UC on placebo (Feagan et al., 2013). In the GEMINI 2 trial, 14.5% of people with CD who were treated with vedolizumab achieved clinical remission by week 6 compared to 6.8% on placebo (Sandborn et al., 2013). For the maintenance arm, 39.0% of people with CD who received vedolizumab every 8 weeks and 36.4% who received vedolizumab every 4 weeks were in clinical remission at 52 weeks, compared to 21.6% on placebo (Sandborn et al., 2013). In the GEMINI 3 trial, vedolizumab was subsequently shown to be effective for people who have moderate to severe CD and are refractory to TNF antagonists, but induction of remission required 10 weeks of treatment (Sands et al., 2014). Based on this, in May 2014, the FDA and European Medicines Agency (EMA) approved vedolizumab for the treatment of UC and CD (Raine, 2014).

## Vedolizumab: Potential Mechanisms of Action

Vedolizumab was designed to reduce intestinal inflammation by interfering with the T-cell trafficking to the intestines (Picarella et al., 1997; Feagan et al., 2013). As mentioned earlier, α4β7 integrin is a receptor expressed on lymphocytes that recognizes MAdCAM-1 (Berlin et al., 1993). MAdCAM-1 is constitutively expressed on venular endothelium and upregulated during inflammation (Briskin et al., 1997). Though already approved by the FDA for treatment of UC and CD, the molecular mechanisms of vedolizumab in humans have not been elucidated and still require further study.

In a study by Zeissig et al. (2019), T-cell trafficking to the intestinal lamina propria in people with CD and UC, who were treated with vedolizumab, was not reduced. Vedolizumab treatment did not affect the intestinal T-cell receptor repertoire or the relative abundance of lamina propria CD4+ T-cells, CD8+ T-cells, central memory T-cells, or effector memory T-cells. However, *in vitro* and *in vivo* models of intestinal inflammation have been used to investigate the potential effects of vedolizumab on T-cell migration to the intestine (Fischer et al., 2016; Binder et al., 2018). An inventive *in vitro* model of blood flow using glass tubes and a peristaltic pump to mimic blood flow through capillaries was highlighted by Binder et al. (2018). The model allowed for researchers to study the effects of vedolizumab treatment on integrin adhesion properties and expression in various T-cell subsets in a distinct, controlled environment (Binder et al., 2018). The group reported that vedolizumab treatment reduced the adhesion of CD4+ and CD8+ T-cells to MAdCAM-1 (Binder et al., 2018). Fischer et al. (2016) injected human T-cells or PBMCs into the ileocolic artery of mice lacking murine lymphocytes and NK cells. They demonstrated that vedolizumab specifically restricts the migration of Tregs from people with UC in this model but does not affect the migration of effector T-cells (Fischer et al., 2016). In contrast, Lord et al. (2018) reported differences in α4β7 expression on circulating lymphocytes, postulating that vedolizumab may preferentially block the recruitment of pro-inflammatory cells to the intestine. Moreover, because Tregs express less α4β7 than effector cells, they may be less affected by vedolizumab and successfully recruited to inflammatory sites where they suppress local inflammation (Lord et al., 2018). *Ex vivo* work with blood and colonic biopsies from people with IBD, published by Rath et al. (2018), suggests that vedolizumab treatment reduces α4β7 integrin expression on B cells, NK cells, Th1, Th2, and Th17 CD4+ T-cell subsets. In addition, vedolizumab therapy-induced clinical remission was associated with a reduction of α4β7 expression on Th2 and Th17 mucosal CD4+ T-cells, which together could reduce recruitment of pro-inflammatory cells to the gut mucosa. Moreover, Rath et al. (2018) suggested that higher α4β7 expression on T-cells before vedolizumab treatment was associated with clinical remission. Together these studies suggest that vedolizumab may act *via* selective inhibition of specific T-cell subtypes migrating to the gut.

Clahsen et al. (2015) reported the CD103+ (a subunit of the αEβ7 integrin) subpopulation of cDCs is reduced in MAdCAM-1 deficient mice. CD103+ cDCs play a role in inducing the expression of α4β7 integrin on T-cells and promoting Treg cell development. This may explain the observation made by Fischer et al. (2016) that Treg cell migration was reduced by vedolizumab. If vedolizumab treatment similarly results in fewer CD103+ cDCs, α4β7 expression would be reduced, limiting T-cell recruitment. Fuchs et al. (2017) found an association of αEβ7 expression on effector T-cells with worsened clinical parameters. They propose that αEβ7 upregulation may be an alternative pathway for lymphocytes beyond the α4β7-MAdCAM-1 axis (Fuchs et al., 2017). Furthermore, Zundler et al. (2017) reported that αEβ7 expression was increased on CD8+ T-cells following vedolizumab treatment in people with IBD, suggesting that lymphocytes may use an alternative integrin to ensure their localization in the intestine despite vedolizumab-mediated inhibition of α4β7. Together, this suggests that vedolizumab may indeed cause changes in T-cell recruitment. Though results of these studies suggest that vedolizumab restricts T-cell recruitment *via* integrin binding, there are limitations in the applicability of *in vitro* and *in vivo* animal models when studying vedolizumab, and additional work is required to translate these findings for people with IBD.

**Figure 1:**
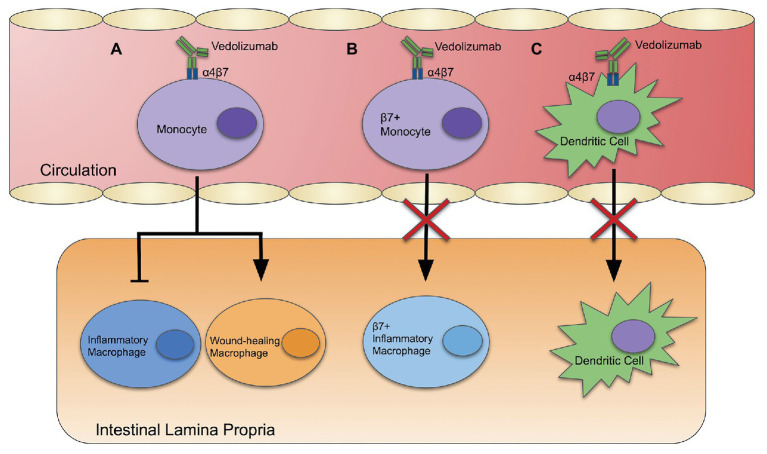
Vedolizumab: three potential mechanisms of action. **(A)** Vedolizumab binds α4β7 integrin, which alters gene expression of blood monocytes, skewing the population toward a wound-healing phenotype, and away from an inflammatory phenotype. **(B)** Vedolizumab binds to α4β7 integrin on blood monocytes, thereby inhibiting their ability to enter the intestinal epithelium. **(C)** Vedolizumab blocks localization of cDC and pDCs in the intestinal epithelium by binding α4β7.

Alternative mechanisms of action in blocking monocyte and dendritic cell recruitment have been proposed (Figure 1). Zeissig et al. (2019) investigated the migration of leukocytes to the intestines by staining and labeling peripheral blood leukocytes with Indium-111 and fluorescein for scintigraphy and endomicroscopy. Imaging showed the accumulation of leukocytes in the intestines was not affected by vedolizumab. However, there was a strong association between vedolizumab treatment and the downregulation of genes involved in the innate immune system in the sigmoid colon. Genes that regulate innate effectors, innate immune receptors, chemokines, and chemokine receptors were downregulated in people with UC and CD who achieved clinical remission with vedolizumab. Interestingly, the relative abundance of inflammatory and wound-healing macrophages was also affected by vedolizumab treatment, skewing macrophages toward a healing phenotype (Figure 1A).

Conversely, vedolizumab treatment has also been reported to interfere with homing of non-classical monocytes, which skew toward wound-healing macrophages (Olingy et al., 2017; Schleier et al., 2020). Approximately 5% of non-classical monocytes express α4β7 integrin, so it has been suggested that vedolizumab may actually disrupt intestinal wound healing and lead to complications (Schleier et al., 2020). Despite that, Danese et al. (2019) found that people with moderate to severe CD who were treated with vedolizumab presented with endoscopic and histological healing at both 26 and 52 weeks. Furthermore, Shen et al. (2019) analyzed data from the GEMINI 1, GEMINI 2, GEMINI Long Term Safety studies, and Vedolizumab Global Safety Database, reporting only a minor difference in the frequency of postoperative complications after intestinal surgery in people treated with vedolizumab compared to placebo. Together, this suggests that the beneficial anti-inflammatory effects of vedolizumab may override concerns about a lack of recruitment of non-classical monocytes, compromised wound healing, and downstream complications.

Schippers et al. (2016) suggested that the therapeutic efficacy of vedolizumab is linked to changes in the innate immune system rather than the adaptive immune system. They reported that the β7-integrin chain leads to recruitment of more inflammatory monocytes to the colon in the dextran sodium sulfate (DSS) model of IBD. In this model, DSS is administered in the drinking water of mice to disrupt the intestinal epithelial layer. The compromised intestinal barrier allows the luminal microbes to interact with underlying immune cells, leading to colitis that models human UC. β7-integrin deficient mice had a lower disease activity index (DAI; an additive score based on weight loss, stool consistency, and fecal blood). Colonic mRNA expression of inducible nitric oxide synthase (iNOS), proinflammatory cytokines, such as IL-6 and TNFα, and the chemokine CCL2 was lower in β7-integrin deficient mice than wild-type mice. RAG2 deficient mice, which lack mature T and B cells (Shinkai et al., 1992), were more susceptible to DSS-induced colitis than their wild-type counterparts. β7-integrin deficiency protected DSS-treated RAG2 deficient mice and mice double deficient in β7 and RAG2 in DAI and histopathology (Schippers et al., 2016). This suggests that an anti-integrin therapy, like vedolizumab, may block the recruitment of proinflammatory monocytes to the site of inflammation (Figure 1B).

Mucosal addressin cellular adhesion molecule primarily interacts with the α4β7 integrin to mediate lymphocyte homing, including effector and memory T-cells. Clahsen et al. (2015) propose a novel role for MAdCAM-1 in mediating intestinal localization of DCs to the intestinal epithelium. They found that MAdCAM-1 deficient mice and β7-integrin deficient mice had lower numbers of cDCs and pDCs in the intestinal epithelium compared to wild-type mice. They propose that MAdCAM-1 may mediate localization of cDCs and pDCs into the gut *via* the α4β7 integrin. Thus, vedolizumab may work, in part, by blocking recruitment of DCs that promote inflammation (Figure 1C).

## Future Directions

A long-term goal including a system wide analysis of the effect of vedolizumab on α4β7- and αEβ7-mediated trafficking, myeloid and T-cell populations in the intestine, and clinical outcomes would be of tremendous value in this field. Additionally, the possibility that selective T-cell trafficking is blocked by vedolizumab should continue to elucidate the effect of less abundant T-cell populations, like Tregs, and the important ratio of Tregs/effector T-cells in the gut after vedolizumab treatment. Vedolizumab may also work in part by blocking pro-inflammatory monocyte and dendritic cell recruitment. Further studies on the properties of leukocyte populations in relation to vedolizumab treatment for people with IBD should be conducted. Additional studies using data derived from people with IBD being treated with vedolizumab are essential to translate potential mechanisms elucidated *in vitro* and in murine models to people with IBD.

## Conclusion

In this paper, we reviewed multiple proposed mechanisms of action for vedolizumab, a relatively new biologic used to treat IBD. It was specifically developed to block T-cell trafficking to the gut, but recent evidence suggests that this may not be its sole mechanism of action. Three alternative mechanisms of action have been reported. Zeissig et al. (2019) examined a mechanism where vedolizumab intervention led to the downregulation of inflammatory gene expression in innate immune cells like monocytes. Clahsen et al. (2015) described a mechanism in which the MAdCAM-1 interaction with α4β7 integrin of pDCs and cDCs is necessary for migration into the intestinal epithelium, where these dendritic cells have been known to exhibit pro-inflammatory phenotypes. Lastly, Schippers et al. (2016) demonstrated a mechanism whereby vedolizumab restricts the recruitment of β7+ effector monocytes to the intestinal epithelium, thereby limiting the inflammatory response. Understanding the mechanism(s) of action of vedolizumab may enable us to improve the efficacy of current treatment and the develop new therapeutic strategies to treat IBD.

## Author Contributions

This concept for this work was created by LMS with contributions from ML-S, YP, and AZ. ML-S, YP, and AZ contributed equally to manuscript preparation. Figures were prepared by JS with contributions from ML-S, YP, AZ, and LMS. All authors contributed to the article and approved the submitted version.

### Conflict of Interest

The authors declare that the research was conducted in the absence of any commercial or financial relationships that could be construed as a potential conflict of interest.
